# Supplementary material for: Diet and gut microbiome enterotype are associated at the population level in African buffalo
Source: Nat Commun. 2021 Apr 15;12:2267. doi: 10.1038/s41467-021-22510-8 (PMC8050287; doi:10.1038/s41467-021-22510-8)
Supplement: Supplementary file 1 — Supplementary Information [file 41467_2021_22510_MOESM1_ESM.pdf]

Supplementary Materials

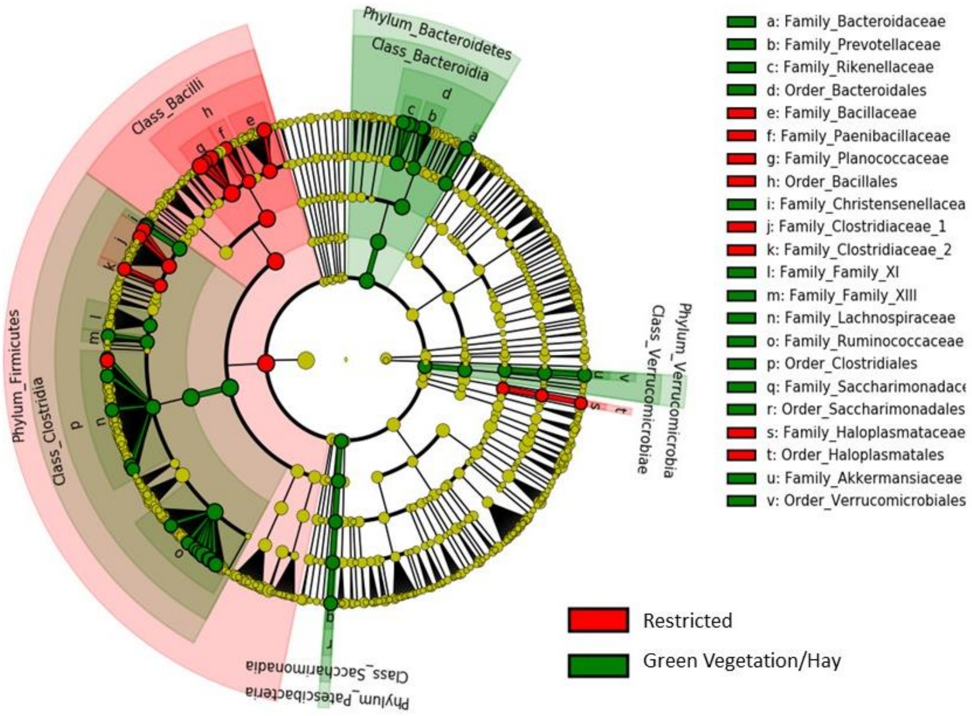

Supplementary Figure S1: Linear discriminate analysis effect size (LEfSe) results demonstrate differentially abundant taxa between the high nutrition regimes (green vegetation/hay) versus the restricted feed regime. Differentially abundant taxa with an LDA score of at least 3.5 (log 10) are shown from the phylum through family levels. Order and family levels are abbreviated for clarity on the cladogram and are shown in the legend. In this analysis, classes were defined as high nutrition (hay or green vegetation) versus the restricted, while subclasses were defined by specific diet regime (hay, green vegetation, restricted).

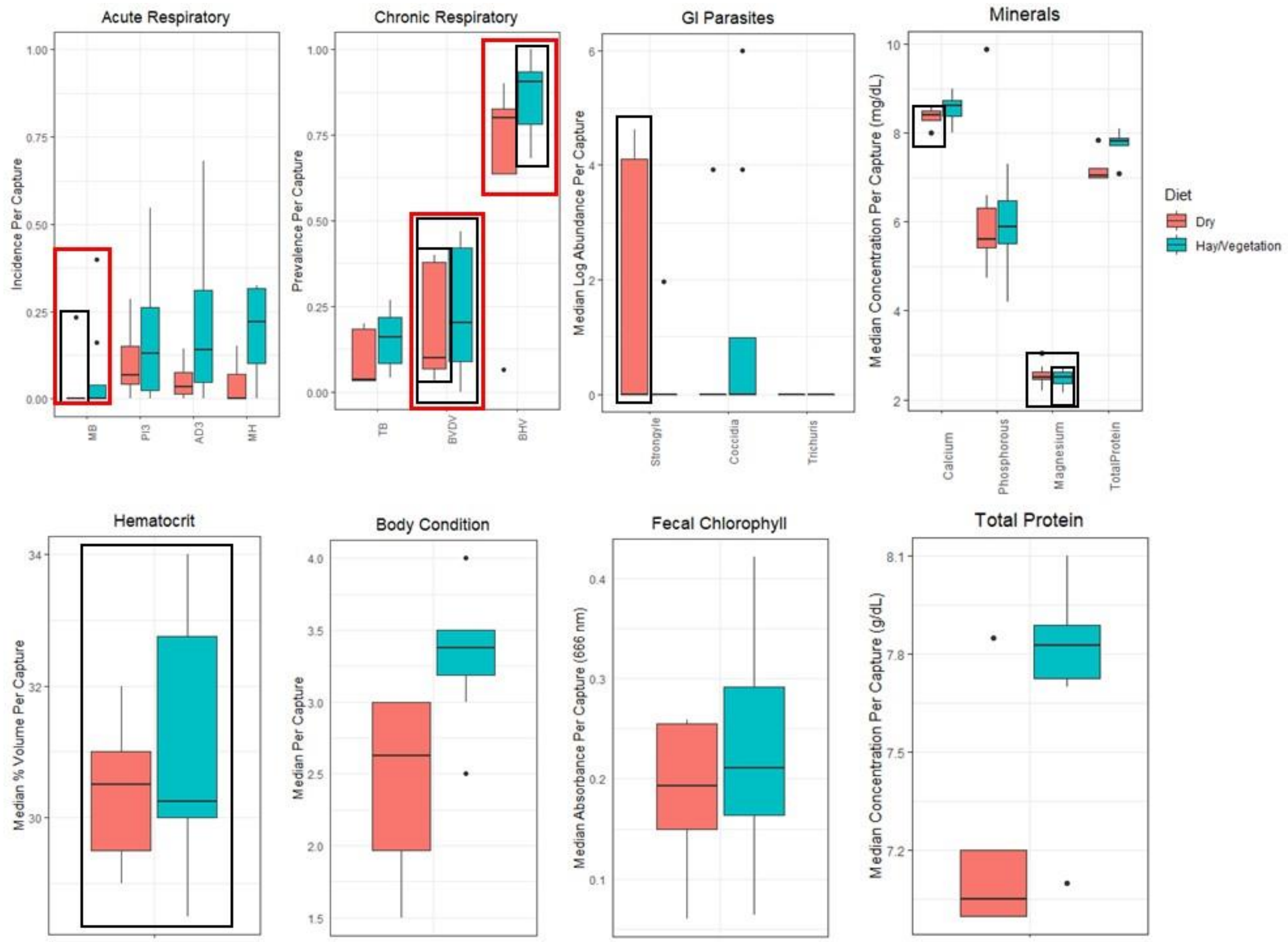

Supplementary Figure S2: Distributions of covariates included in microbiome compositional analysis. Summary values were calculated for each capture (incidence, prevalence, or median values), and summary statistics of were plotted to show between-capture variation. Values were calculated for 15 separate time points from a total of 426 individual samples. Incidence was defined for *Mycoplasma bovis* (MB), parainfluenza-3, adenovirus-3, and *Manheimia hemolytica* as percent of animals who seroconverted from negative to positive between captures. Prevalence of bovine tuberculosis (TB), bovine viral diarrhea virus (BVDV) and bovine herpes virus (BHV) was defined as percent of animals who tested positive at each capture. For each variable, the horizontal bar shows the median value, the lower and upper hinges correspond to the first and third quartiles, the lower and upper whiskers correspond to the smallest and largest values at most 1.5 * IQR of the hinge. Data outside this range was plotted as individual points. Variables are outlined in black if the initial envfit analysis showed significant correlations with microbiome composition in the dry or hay/vegetation regimes or the pooled data. Variables are outlined in red if they were significant in the final CCA model following model selection. Source data are provided as a Source Data file.

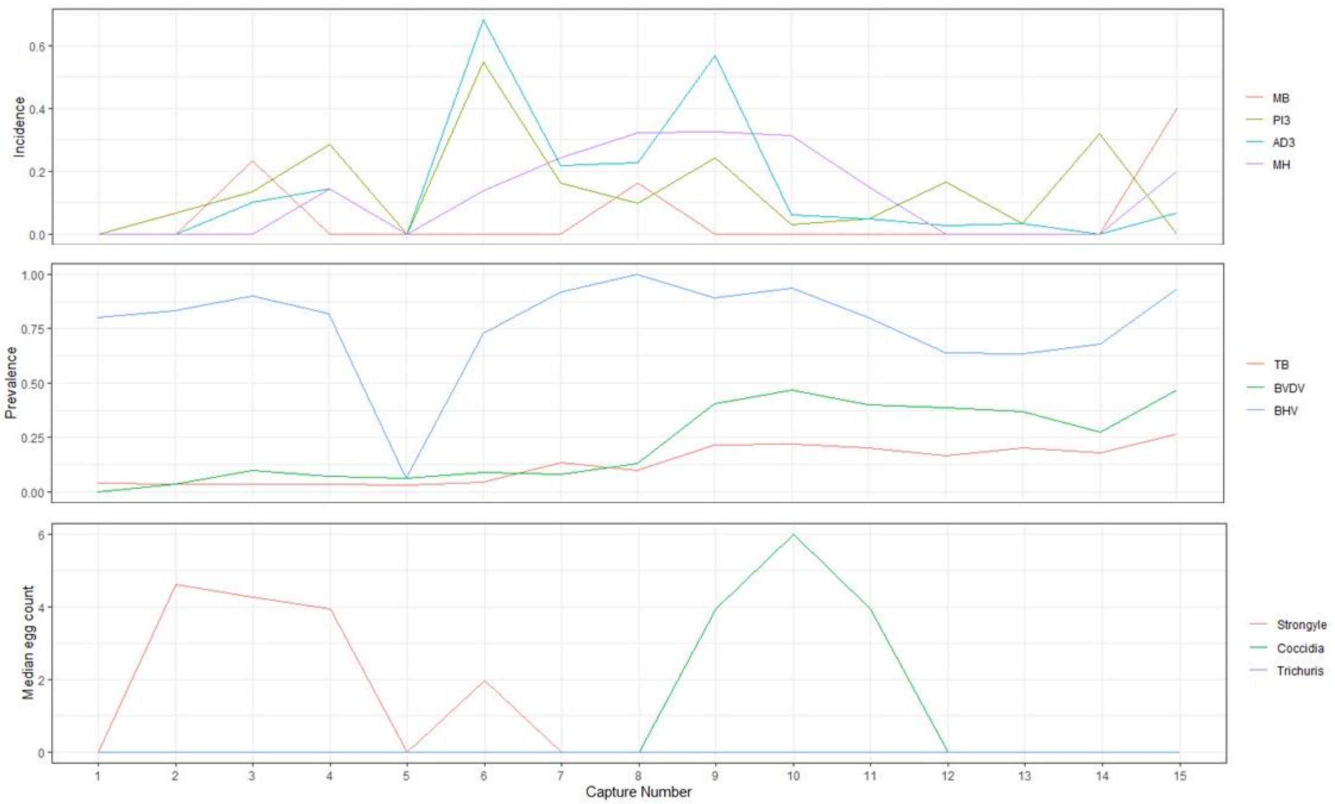

Supplementary Figure S3: Changes in infection over the course of the study. Incidence of acute respiratory infections (top), prevalence of chronic respiratory infections (middle), and median gastrointestinal parasite egg counts for each capture period throughout the study. Incidence was defined for *Mycoplasma bovis* (MB), parainfluenza-3, adenovirus-3, and *Manheimia hemolytica* as percent of animals who seroconverted from negative to positive between captures. Prevalence of bovine tuberculosis (TB), bovine viral diarrhea virus (BVDV) and bovine herpes virus (BHV) was defined as percent of animals who tested positive at each capture.

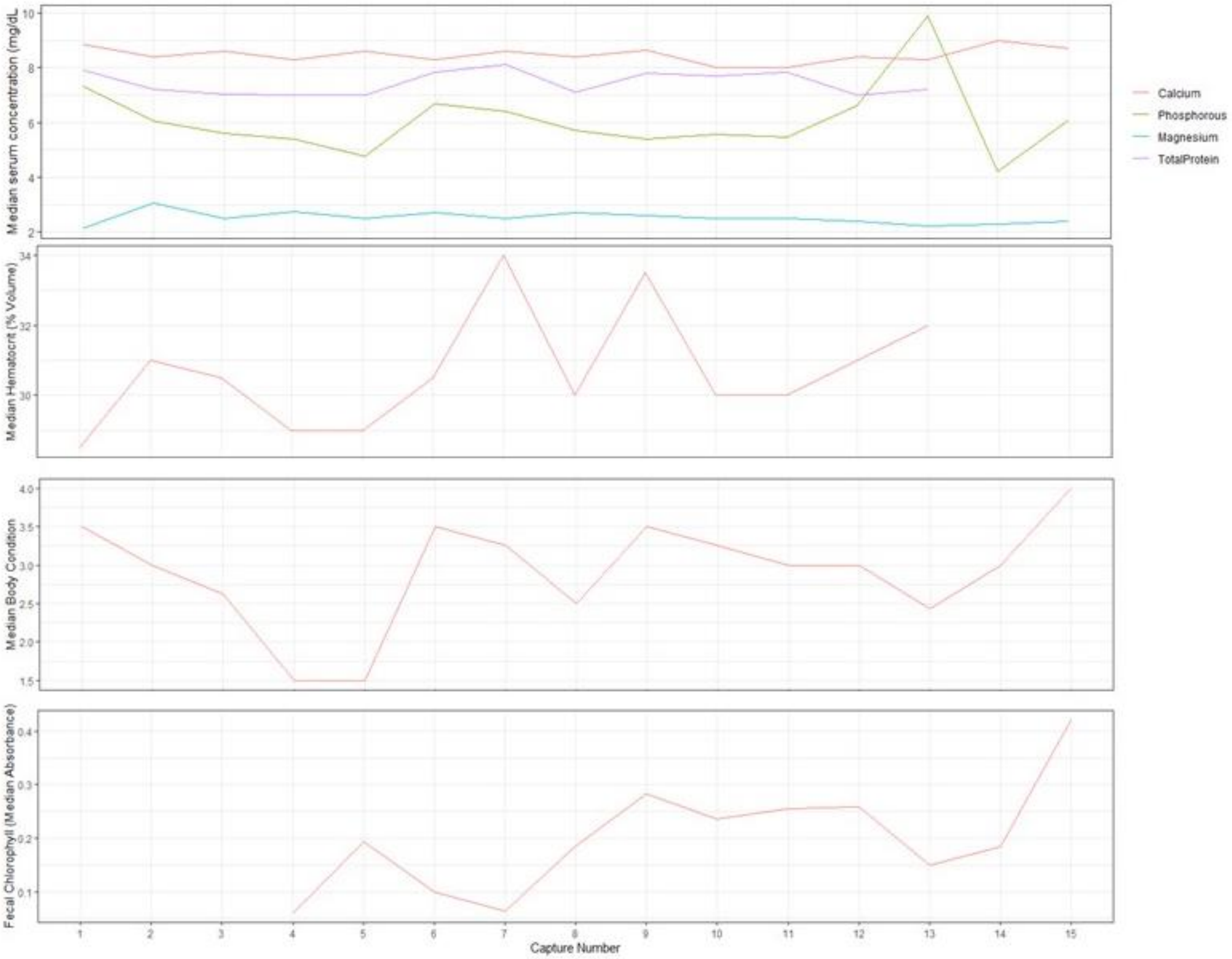

Supplementary Figure S4: Changes in nutritional parameters over the course of the study. The top panel shows changes in serum concentrations of calcium, phosphorus, magnesium, and total protein (mg/dL). The panel second from the top shows changes in median hematocrit values, which were only measured up to capture 13. The panel second from the bottom shows changes in mean body condition score, which was measured on a scale from 1-4. The bottom panel illustrates changes in median fecal chlorophyll, measured by absorbance at 666 nm. Chlorophyll was not measured for the first three captures.
