# Supplementary material for: Diet and gut microbiome enterotype are associated at the population level in African buffalo
Source: Nat Commun. 2021 Apr 15;12:2267. doi: 10.1038/s41467-021-22510-8 (PMC8050287; doi:10.1038/s41467-021-22510-8)
Supplement: Supplementary file 3 — Reporting Summary [file 41467_2021_22510_MOESM3_ESM.pdf]

nature research

Corresponding author(s): Claire Couch
Last updated by author(s): Feb 13, 2021

Reporting Summary

Nature Research wishes to improve the reproducibility of the work that we publish. This form provides structure for consistency and transparency in reporting. For further information on Nature Research policies, see our Editorial Policies and the Editorial Policy Checklist.

Statistics

For all statistical analyses, confirm that the following items are present in the figure legend, table legend, main text, or Methods section.

n/a	Confirmed	
☐	☒	The exact sample size (*n*) for each experimental group/condition, given as a discrete number and unit of measurement
☐	☒	A statement on whether measurements were taken from distinct samples or whether the same sample was measured repeatedly
☐	☒	The statistical test(s) used AND whether they are one- or two-sided<br>*Only common tests should be described solely by name; describe more complex techniques in the Methods section.*
☐	☒	A description of all covariates tested
☐	☒	A description of any assumptions or corrections, such as tests of normality and adjustment for multiple comparisons
☐	☒	A full description of the statistical parameters including central tendency (e.g. means) or other basic estimates (e.g. regression coefficient) AND variation (e.g. standard deviation) or associated estimates of uncertainty (e.g. confidence intervals)
☐	☒	For null hypothesis testing, the test statistic (e.g. *F*, *t*, *r*) with confidence intervals, effect sizes, degrees of freedom and *P* value noted<br>*Give P values as exact values whenever suitable.*
☒	☐	For Bayesian analysis, information on the choice of priors and Markov chain Monte Carlo settings
☒	☐	For hierarchical and complex designs, identification of the appropriate level for tests and full reporting of outcomes
☐	☒	Estimates of effect sizes (e.g. Cohen's *d*, Pearson's *r*), indicating how they were calculated

Our web collection on statistics for biologists contains articles on many of the points above.

Software and code

Policy information about availability of computer code

Data collection	Miseq control software v2.6.2.1
Data analysis	R version 3.6.3; R packages: raster (version 3.3.13), sp (version 1.4.2), rgdal (version 1.5.16), maptools (version 1.0.1), rgeos (version 0.5.3), phyloseq (version 1.30.0), metacoder (version 0.3.4), vegan (version 2.5.6), dada2 (version 1.14.1), lme4 (version 1.1.21), cluster (version 2.1.0), rptR (version 0.9.22), cplm (version 0.7.8); LefSE galaxy interface (version 1, https://huttenhower.sph.harvard.edu/galaxy/)

For manuscripts utilizing custom algorithms or software that are central to the research but not yet described in published literature, software must be made available to editors and reviewers. We strongly encourage code deposition in a community repository (e.g. GitHub). See the Nature Research guidelines for submitting code & software for further information.

Data

Policy information about availability of data

All manuscripts must include a data availability statement. This statement should provide the following information, where applicable:

- Accession codes, unique identifiers, or web links for publicly available datasets
- A list of figures that have associated raw data
- A description of any restrictions on data availability

Microbiome sequence data are available in the NCBI SRA database (BioProject ID PRJNA694651). MODIS for NACP data is available at https://accweb.gsfc.nasa.gov/. All other datasets generated during and/or analysed during the current study are available from the corresponding author on reasonable request.

Field-specific reporting

Please select the one below that is the best fit for your research. If you are not sure, read the appropriate sections before making your selection.

☐ Life sciences ☐ Behavioural & social sciences ☒ Ecological, evolutionary & environmental sciences

For a reference copy of the document with all sections, see nature.com/documents/nr-reporting-summary-flat.pdf

Ecological, evolutionary & environmental sciences study design

All studies must disclose on these points even when the disclosure is negative.

Study description

A herd of 60-70 African buffalo (depending on births and deaths) was longitudinally studied to assess changes in fecal microbiota associated with diet, disease, and physiology. Diet was catagorically defined based on NDVI and supplemental feed. Disease was measured by commercial ELISA kits. Physiology was measured using commercial serum biochemistry kits and physical measurements.

Research sample

A herd of male and female African buffalo (Syncerus caffer) ranging in age from 0 to 19 years (estimated)was followed as part of a concurrent study on foot and mouth disease virus. The age and sex distribution of the herd depended on births and deaths throughout the study. The herd was located in a 900 hectare enclosure in Kruger National Park, South Africa. The enclosure was designed to exclude large predators, but included other herbivore species and small predators. The herd was selected to represent a naturally-occurring buffalo population in Kruger National Park for the purpose of studying disease transmission. Large predators were excluded to prevent predation of diseased buffalo. As a proxy for vegetation and dietary availability, we used 16-day composite, 250-m resolution NDVI data from MODIS for the North American Carbon Program (MODIS for NACP, https://accweb.gsfc.nasa.gov/).

Sampling strategy

Every 2-4 months, we captured each animal in the study herd. As part of the concurrent disease study, it was necessary to sample all individuals to assess FMDV spread, so we opportunistically obtained convenience fecal samples from all individuals captured.

Data collection

Fecal samples were collected directly from each buffalo by hand using a sterile glove and inverting into a whirl pack. Fecal samples were placed in ice immediately after capture until transport back to the lab, where they were frozen at -80 C. Blood was collected via jugular venipuncture with (plasma, whole blood) or without (serum) heparin, and stored on ice for transport back to the laboratory. Immediately upon arrival at the laboratory, blood was centrifuged at 5000×g for 10min; plasma and serum pipetted off the cellular layer into sterile microcentrifuge tubes and stored at −80°C until analysis. Serum biochemistry parameters were measured using the Abaxis Vetscan VS2 (Abaxis Inc., Union City, CA, USA) chemistry analyzer and the large animal profile (Abaxis SKU500-023). Sandwich ELISAs were used to measure seroconversion of the following respiratory pathogens per manufacturers' instructions Adenovirus (AD-3), parainfluenza virus (Pi-3), bovine herpes virus, Mannheimia hemolytica, Mycoplasma bovis (MB) (Bio-X IPAMM); bovine diarrhea virus (BVDV) (Bio-X BVDV); bovine respiratory syncytial virus (Bio-X BRSV), Bovine tuberculosis (Thermo Fisher BOVIGAM). Graduate students and technicians from the research group of Dr. Anna Jolles were responsible for processing blood and fecal samples and running disease assays.

Timing and spatial scale

This study uses data collected between February 2014-December 2016, with time points distributed every 2-4 months. There is a gap in sampling between August and December 2015 due to missing fecal samples from the October 2015 capture. Timing for sample collection, as well as the start and end points, was determined by the needs of the concurrent FMDV study which was designed to assess disease transmission dynamics. All fecal samples were collected from buffalo that were kept in a 900 hectare enclosure in Kruger National Park, South Africa.

Data exclusions

Microbiome data were excluded from analyses only if relevant covariates were unavailable for the analysis under consideration. For the CCA
analysis, only samples with associated data for all covariates of interest were included in the analysis.

Reproducibility

Sample collection and storage methods were consistent across the study period. Respiratory ELISAs were performed in duplicate or triplicate, according to the manufacterers' instructions. ELISA results were included only if attempts at replication were successful. Serum biochemistry panels were replicated only once, due to the cost of running the panels. The VetScan we used was serviced twice per year, and is equipped with intelligent quality control to verify chemistry, optics, and electronic functions of the analyzer during each run. Chemical methods, including sensitivity and specificity for each parameter in the large animal profile, are described by the manufacturer (VetScan Large Animal Profile Product Information. Union City, California: Abaxis, Inc; 2001). Amplification PCR was performed only once per sample due to time and cost constraints, however we corrected for amplification bias by subsampling sequencing reads to an even depth. Fecal DNA samples were retained in order to facilitate future replication of the study.

Randomization

No randomization was performed, as this was not an experimental study. Variation explained by animal age, sex, individual ID, and diet were controlled for where applicable.

Blinding

Not applicable, as this was an exploratory/descriptive study. Biological data collection occurred prior to the design of the microbiome study.

Did the study involve field work? ☒ Yes ☐ No

Field work, collection and transport

Field conditions

Fieldwork was conducted in all seasons, under seasonal conditions typical of northeastern South Africa. Temperature and rainfall

Field conditions	were not directly recorded in this study.
Location	The buffalo in this study were located in an enclosure adjacent to Satara rest camp (24.3930° S, 31.7798° E) in Kruger National Park, South Africa. The enclosure is approximately 266 m above mean sea level and has no permanent water features.
Access & import/export	Biological sample processing was carried out at our field site in South Africa under permits from Oregon State University and South Africa National Parks. DNA samples were shipped to Oregon State University following DNA extraction. This study was approved by the institutional animal care and use committee at Oregon State University, ACUP project number#4478, and by Kruger National Park, ACUP project number JOLAE1157-12.
Disturbance	This study took advantage of a concurrent disease research project in the African buffalo population, and as such did not cause any additional disturbance beyond what was necessary for the disease study. For the disease study, disturbance was minimized by acclimating buffalo to vehicle and human presence in order to reduce capture stress.

Reporting for specific materials, systems and methods

We require information from authors about some types of materials, experimental systems and methods used in many studies. Here, indicate whether each material, system or method listed is relevant to your study. If you are not sure if a list item applies to your research, read the appropriate section before selecting a response.

Materials & experimental systems

n/a	Involved in the study	
☒	☐	Antibodies
☒	☐	Eukaryotic cell lines
☒	☐	Palaeontology and archaeology
☐	☒	Animals and other organisms
☒	☐	Human research participants
☒	☐	Clinical data
☒	☐	Dual use research of concern

Methods

n/a	Involved in the study	
☒	☐	ChIP-seq
☒	☐	Flow cytometry
☒	☐	MRI-based neuroimaging

Animals and other organisms

Policy information about studies involving animals; ARRIVE guidelines recommended for reporting animal research

Laboratory animals	No laboratory animals were used in this study.
Wild animals	The herd of African buffalo (Syncerus caffer) in this study has been maintained in the same emi-wild setting for over 15 years for research purposes. For this study, male and female African buffalo ranging in age from 0-19 years (approximate) were sedated with a high potency opioid (Thianil or etorphine hydrocholoride) and azaperone at dosages appropriate to weight and sex (Kock, M., Meltzer, D. & Burroughs, R. Chemical and physical restraint of wild animals: a training and field manual for African species. IWCS, 2006) . Animals were not transported for this study as they were sampled at the location of sedation. Following the study, animals were retained in the enclosure and continued to be managed by SANParks.
Field-collected samples	Frozen blood and fecal samples are housed in -80 degree freezers with minimal light exposure maintained by Kruger National Park Veterinary Wildlife Services in Skukuza, South Africa. DNA was extracted from frozen samples at the same location, and extracted DNA was shipped to Oregon State University, where DNA samples are currently stored at -80 degrees in a BSL-2 laboratory. All biological samples and DNA extracts will be archived indefinitely.
Ethics oversight	All animal work for this study was approved by the institutional animal care and use committee at Oregon State University, ACUP project number#4478, and by Kruger National Park, ACUP project number JOLAE1157-12.

Note that full information on the approval of the study protocol must also be provided in the manuscript.
